# Cathepsin B Expression and the Correlation with Clinical Aspects of Oral Squamous Cell Carcinoma

**DOI:** 10.1371/journal.pone.0152165

**Published:** 2016-03-31

**Authors:** Wei-En Yang, Chuan-Chen Ho, Shun-Fa Yang, Shu-Hui Lin, Kun-Tu Yeh, Chiao-Wen Lin, Mu-Kuan Chen

**Affiliations:** 1 Institute of Medicine, Chung Shan Medical University, Taichung, Taiwan; 2 Department of Otorhinolaryngology-Head and Neck Surgery, Changhua Christian Hospital, Changhua, Taiwan; 3 Department of Medical Research, Chung Shan Medical University Hospital, Taichung, Taiwan; 4 School of Dentistry, Chung Shan Medical University, Taichung, Taiwan; 5 Department of Dentistry, Chung Shan Medical University Hospital, Taichung, Taiwan; 6 Department of Surgical Pathology, Changhua Christian Hospital, Changhua, Taiwan; 7 Department of Medical Technology, Jen-Teh Junior College of Medicine, Nursing and Management, Miaoli, Taiwan; 8 Institute of Oral Sciences, Chung Shan Medical University, Taichung, Taiwan; Sapporo Medical University, JAPAN

## Abstract

**Background:**

Cathepsin B (CTSB), a member of the cathepsin family, is a cysteine protease that is widely distributed in the lysosomes of cells in various tissues. It is overexpressed in several human cancers and may be related to tumorigenesis. The main purpose of this study was to analyze CTSB expression in oral squamous cell carcinoma (OSCC) and its correlation with patient prognosis.

**Methodology/Principal Findings:**

Tissue microarrays were used to detect CTSB expression in 280 patients and to examine the association between CTSB expression and clinicopathological parameters. In addition, the metastatic effects of the CTSB knockdown on two oral cancer cell lines were investigated by transwell migration assay. Cytoplasmic CTSB expression was detected in 34.6% (97/280) of patients. CTSB expression was correlated with positive lymph node metastasis (p = 0.007) and higher tumor grade (p = 0.008) but not with tumor size and distant metastasis. In addition, multivariate analysis using a Cox proportional hazards model revealed a higher hazard ratio, demonstrating that CTSB expression was an independent unfavorable prognostic factor in buccal mucosa carcinoma patients. Furthermore, the Kaplan–Meier curve revealed that buccal mucosa OSCC patients with positive CTSB expression had significantly shorter overall survival. Moreover, treatment with the CTSB siRNA exerted an inhibitory effect on migration in OC2 and CAL27 oral cancer cells.

**Conclusions:**

We conclude that CTSB expression may be useful for determining OSCC prognosis, particularly for patients with lymph node metastasis, and may function as a biomarker of the survival of OSCC patients in Taiwan.

## Introduction

Oral cancer is the sixth most common cancer worldwide, and approximately 90% of oral cancers are oral squamous cell carcinoma (OSCC) [1]. In Taiwan, oral cancer was the fifth most common cause of death in both sexes and the fourth most common cancer in men in 2014. The risk factors for oral cancer include betel quid chewing, cigarette smoking, alcohol consumption, poor oral health, and human papillomavirus infections [2]. Common treatments for oral cancer include surgery, radiotherapy, or chemotherapy. In recent years, combinations of multidisciplinary approaches have improved the quality of life of OSCC patients, but not the overall 5-year survival rate [3]. Therefore, identifying potential biomarkers to predict cancer progression is crucial.

Cathepsins (CTSs) are multifunctional enzymes that regulate tumor growth, migration, invasion, metastasis, and angiogenesis [4]. Moreover, 12 cysteine CTSs have been identified in humans. Cathepsin B (CTSB), a member of the CTS family, is a lysosomal cysteine protease that is synthesized in the inactive proenzyme form [4]. The maturation process involves the removal of signal peptides at the N-terminus to yield 37-kDa CTSB in lysosomes [5]. The CTSB gene has been mapped to chromosome 8p22 and consists of 13 exons. CTSB may enhance the activity of other proteases, namely matrix metalloproteinase [6], serine protease urokinase plasminogen activator [7], and cathepsin D [8], resulting in extracellular matrix (ECM) component degradation, cell–cell communication disruption, and reduced protease inhibitor expression that mediates the transformation of benign cancers to malignant cancers. Numerous studies have shown that CTSB expression is increased in breast [6], ovarian [9], pancreatic [10], lung [11], and liver cancers [12]. In addition, CTSB is upregulated in premalignant lesions and various pathological conditions, such as tumor invasion, rheumatoid arthritis [13], and osteoarthritis [14]. Notably, CTSB is also involved in autophagic flux in RAW 264.7 macrophages [15]. Overall, CTSB appears to have various roles in cancer cells.

CTSB protein and mRNA levels are increased in OSCC cells, and CTSB promotes both cell invasion and migration [16]. CTSB expression in OSCC has been reported [17]; Yang et al. found that in 30 surgically resected tissue specimens of OSCC patients, higher CTSB protein and mRNA levels were observed in tumor tissues than in adjacent nonmalignant epithelial tissue[17]. However, the clinicopathological characteristics and clinical role of CTSB in OSCC are still unclear. The aim of this study was to evaluate the association between clinicopathological parameters and CTSB in 280 OSCC patients by using immunohistochemistry.

## Materials and Methods

### Patients and tissue microarray

In this study, we collected 280 OSCC patients who underwent treatment at Changhua Christian Hospital, (Changhua, Taiwan) between 2000 and 2006 as previously described [18]. Before commencement of this study, approval was obtained from the Institutional Review Board of Changhua Christian Hospital and informed written consent to participate in the study was obtained from each person.

#### Immunohistochemical Staining

OSCC TMA block slides were deparaffinized in xylene, rehydrated through a series of decreasing dilutions of alcohol and distilled water, and washed with phosphate-buffered saline (PBS) as previously described [19]. The endogenous peroxidase activity was blocked with 3% H2O2. The antigen was retrieved by heating at 100°C for 20 min in 10 mM citrate buffer (pH 6.0). After antigen retrieval, slides were incubated with an anti-Cathepsin B antibody (FL-339 sc-13985, Santa Cruz Biotechnology, Santa Cruz, CA, USA) in a dilution of 1:100x for 30 min at room temperature, and washed three times with PBS. Slides were incubated with an HRP/Fab polymer conjugate for another 30 min. The sites of peroxidase activity were visualized using 3,3'-diamino-benzidine tetrahydrochloride as a substrate. Gill Hematoxylin Solution II (MERCK, Darmstadt, Germany) was utilized as the counterstain. Expression of CTSB was assessed semi-quantitatively based on the staining intensity by two pathologists, who blinded to clinical outcome, scoring coded sections under a light microscope independently. The intensity of staining was scored as negative (score 0), weak (score 1+), and strong (score 2+), respectively.

#### Cell culture

Oral cancer cell line OC2 cells were gifts from Dr. C-C Yu, School of Dentistry, Chung Shan Medical University, Taichung, Taiwan. The CAL27 human oral cancer cell lines were purchased from ATCC (ATCC: American Type Culture Collection, Manassas, VA, USA). OC2 and CAL27 cells were cultured in Dulbecco’s modified Eagle’s medium (Life Technologies, Grand Island, NY, USA). All cell cultures were maintained at 37°C in a humidified atmosphere of 5% CO_2_.

#### Western Blot analysis

Proteins in the total cell lysate (20 μg of protein) were separated by sodium dodecyl sulfate-polyacrylamide gel electrophoresis (SDS-PAGE) in 10% gels and electrotransferred to a polyvinylidene difluoride membrane (Immobilon-P membrane; Millipore, Bedford, MA) as described previously [20]. After the blot was blocked in a solution of 5% skim milk, 0.1% Tween 20, and PBS, membrane-bound proteins were probed with anti-Cathepsin B antibody (FL-339 sc-13985, Santa Cruz Biotechnology, Santa Cruz, CA, USA). The membrane was washed and then incubated with horseradish peroxidase (HRP)-conjugated secondary antibodies for 30 min. Antibody-bound protein bands were detected with enhanced chemiluminescence reagents. Signal was detected by using an enhanced chemi-luminescence (ECL) commercial kit (Amersham Biosciences, Piscataway, NJ, USA).

#### Transwell Migration Assays

After a treatment with the control siRNA and CTSB siRNA (ID: s3739; Applied Biosystems, Foster City, CA, USA) for 48 h, OC2 and CAL27 cells were harvested and assayed using a Transwell chamber (Millipore Corporation, Billerica, MA, USA) at 7x10^4^ cells/well in serum free medium and then incubated for 48 h at 37°C. The migrated cells were fixed with 100% methanol and stained with 5% Giemsa. Cell numbers were counted under a light microscope.

#### Statistical Analysis

Statistical analyses were performed with the SPSS statistical software 17.0 (SPSS Inc., Chicago, IL, USA). Demographic data including age, six, clinical stage, T classification, N classification, M classification, differentiation, death, the continuous variables were presented by mean ± standard deviation; the categorical variables were presented by numbers (%). The Kaplan-Meier method was used to estimate CTSB expression and oral cancer. Survival rates were compared with the log-rank test. Univariate analysis of the independent prognostic factor for survival was performed using the Cox proportional hazard regression model with a 95% confidence interval (CI). A p-value of less than 0.05 was regarded as statistically significant.

## Results

Table 1 presents the clinicopathological characteristics of 280 OSCC patients (265 men and 15 women). Their average age was 55.77 ± 11.10 years. Tumors were distributed in the following locations: buccal mucosa (109), tongue (93), gingival (35), palate (16), floor of the mouth (14), and others (13). The buccal mucosa was the most frequent location of cancer. The tumor stage was classified according to the American Joint Committee on Cancer/Union for International Cancer Control TNM staging system (7th edition); 52 (18.6%) patients had stage I, 56 (20.0%) had stage II, 36 (12.9%) had stage III, and 136 (48.5%) had stage IV. Most tumors were classified as moderately and poorly differentiated tumors.

**Table 1:** Patient characteristics.

Characteristics	Total (%)
**Total number of patients**	**280**
**Age (year)**	
Mean ± SD	55.77 ± 11.10
**Gender**	
Male	265 (94.6%)
Female	15 (5.4%)
**Cancer location**	
Buccal mucosa	109 (38.9%)
Tongue	93 (33.2%)
Gingiva	35 (12.5%)
Palate	16 (5.7%)
Floor of Mouth	14 (5.0%)
Others	13 (4.7%)
**Clinical stage**	
I	52 (18.6%)
II	56 (20.0%)
III	36 (12.9%)
IV	136 (48.5%)
**T classification**	
T1	70 (25.0%)
T2	88 (31.4%)
T3	24 (8.6%)
T4	98 (35.0%)
**N classification**	
N0	177 (63.2%)
N1	36 (12.9%)
N2	63 (22.5%)
N3	4 (1.4%)
**M classification**	
M0	276 (98.6%)
M1	4 (1.4%)
**Grade**	
well	42 (15.0%)
moderate, poor	238 (85.0%)

**Fig 1:**
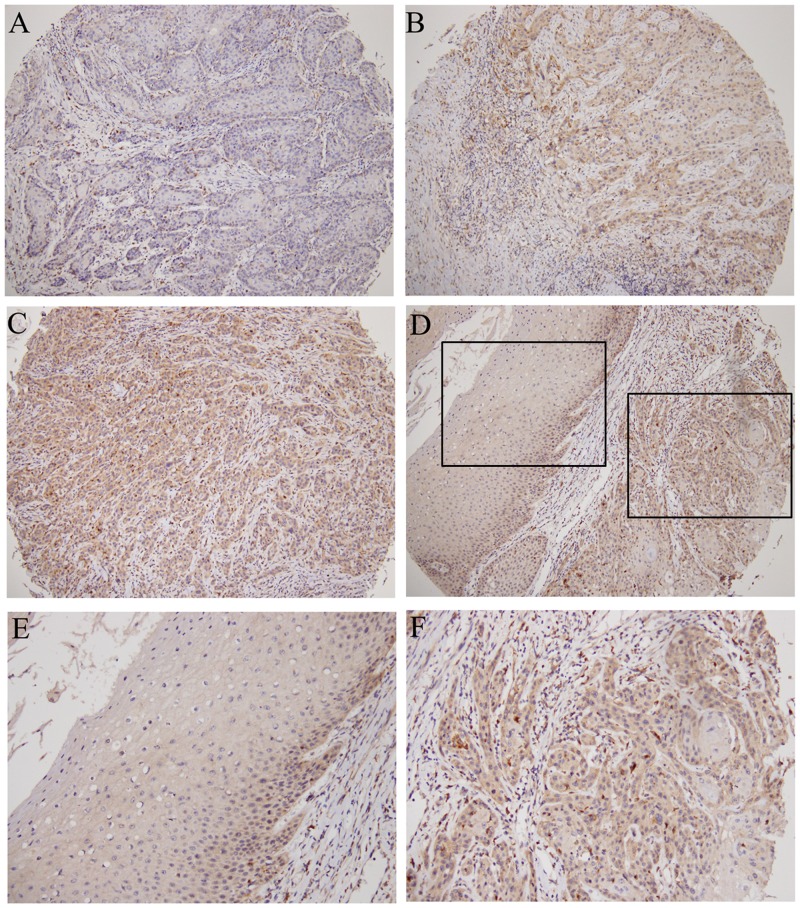
Immunohistochemically staining for the expression of cytoplasmic Cathpesin B (CTSB) in oral cancer. Tissue microarrays of primary oral squamous cell carcinoma (OSCC) (280 cases) were analyzed. (A) No detectable CTSB (0). (B) Weak expression levels (1+). (C) Strong expression levels (2+). (D) The filed had both normal and tumor regions. (E) and (F) show higher magnification of normal and tumor region in the black boxed area of (D) respectively. A–D Low-power field (×100). E and F High-power field (×200).

Cytoplasmic CTSB expression in oral cancer was examined using immunohistochemistry, and patients were divided into two groups on the basis of CTSB staining: overall negative (0) (Fig 1A) and positive (1+/2+) (Fig 1B and 1C). Fig 1D shows CTSB staining in both tumor and nonmalignant epithelial tissues. CTSB staining was weak in nonmalignant epithelial tissue (Fig 1E) but strong in tumor tissue (Fig 1F). Table 2 demonstrates that 34.6% (97/280) of patients were CTSB positive and 65.4% (183/280) were CTSB negative. No significant differences were observed between CTSB protein levels and the patient’s age, sex, cancer location, clinical stage, tumor size, and distant metastasis. However, patients with positive lymph node metastasis (p = 0.007) and poorly differentiated tumors (p = 0.008) had higher CTSB expression.

**Table 2:** Patient characteristics regarding cytoplasmic cathpesin B expression.

	No. of patients (%)		
Characteristics	Cathpesin B (-)	Cathpesin B (+)	p value
Total number of patients	183 (65.4)	97 (34.6)	
**Age (year)**			
<55	84 (45.9)	56 (57.7)	0.060
≥55	99 (54.1)	41 (42.3)	
**Gender**			
Male	175 (95.6)	90 (92.8)	0.314
Female	8 (4.4)	7 (7.2)	
**Cancer location**			
Buccal mucosa	76 (41.5)	33 (34.0)	0.118
Tongue	56 (30.6)	37 (38.1)	
Gingiva	27 (14.8)	8 (8.2)	
Others	24 (13.1)	19 (19.6)	
**Clinical stage**			
I+II	72 (39.3)	36 (37.1)	0.715
III+IV	111 (60.7)	61 (62.9)	
**T classification**			
T1+T2	99 (54.1)	59 (61.9)	0.280
T3+T4	84 (45.9)	38 (38.1)	
**N classification**			0.007*
N0	126 (68.9)	51 (52.6)	
N1+2+3	57 (31.1)	46 (47.4)	
**M classification**			0.683
M0	180 (98.4)	96 (99.0)	
M1	3 (1.6)	1 (1.0)	
**Grade**			0.008*
well	35 (19.1)	7 (7.2)	
moderate, poor	148 (80.9)	90 (92.8)	

*p<0.05

Univariate and multivariate analyses using a Cox proportional hazards model were employed to examine the association between CTSB expression and clinicopathological parameters (Table 3). In all OSCC patients, univariate and multivariate analyses revealed a higher hazard ratio for the tumor size (p < 0.05) and lymph node metastasis (p < 0.001). Moreover, CTSB expression (p = 0.047) was identified as an independent unfavorable prognostic factor in buccal mucosa SCC patients.

**Table 3:** Univariate and multivariate analysis of Cathepsin B and clinicopathological parameters among patients with oral cancer using the Cox proportional hazard regression model.

**All cases (N = 280)**	**Univariate**		**Multivariate**	
	**Hazard ratio (95% CI)**	**p value**	**Hazard ratio (95% CI)**	**p value**
Clinical stage (stage 1 + 2 versus stage 3 + 4)	3.130 (1.898–5.161)	< 0.001*	0.724 (0.254–2.067)	0.546
T status (T1 + T2 versus T3 + T4)	2.162 (1.329–3.519)	0.002*	2.436 (1.007–5.893)	0.048*
N status (N0 versus N1 + N2 + N3)	4.477 (2.594–7.724)	< 0.001*	4.582 (2.011–10.441)	< 0.001*
Grade (Well versus moderate + poor)	2.242 (1.143–4.399)	0.014*	1.850 (0.902–3.792)	0.093
Cathepsin B (+ versus -)	1.535 (0.930–2.536)	0.094	1.278 (0.735–2.222)	0.386
**Buccal mucosa (N = 109)**	**Univariate**		**Multivariate**	
	**Hazard ratio (95% CI)**	**p value**	**Hazard ratio (95% CI)**	**p value**
Clinical stage (stage 1 + 2 versus stage 3 + 4)	3.513 (1.592–7.751)	0.002	0.475 (0.073–3.105)	0.437
T status (T1 + T2 versus T3 + T4)	2.054 (0.913–4.620)	0.082	3.858 (0.682–21.822)	0.127
N status (N0 versus N1 + N2 + N3)	6.963 (2.682–18.075)	< 0.001*	9.528 (1.770–51.291)	0.009*
Grade (Well versus moderate + poor)	2.000 (0.624–6.412)	0.244	1.424 (0.388–5.226)	0.594
Cathepsin B (+ versus -)	3.162 (1.324–7.556)	0.010*	2.732 (1.016–7.349)	0.047*

*p<0.05

**Fig 2:**
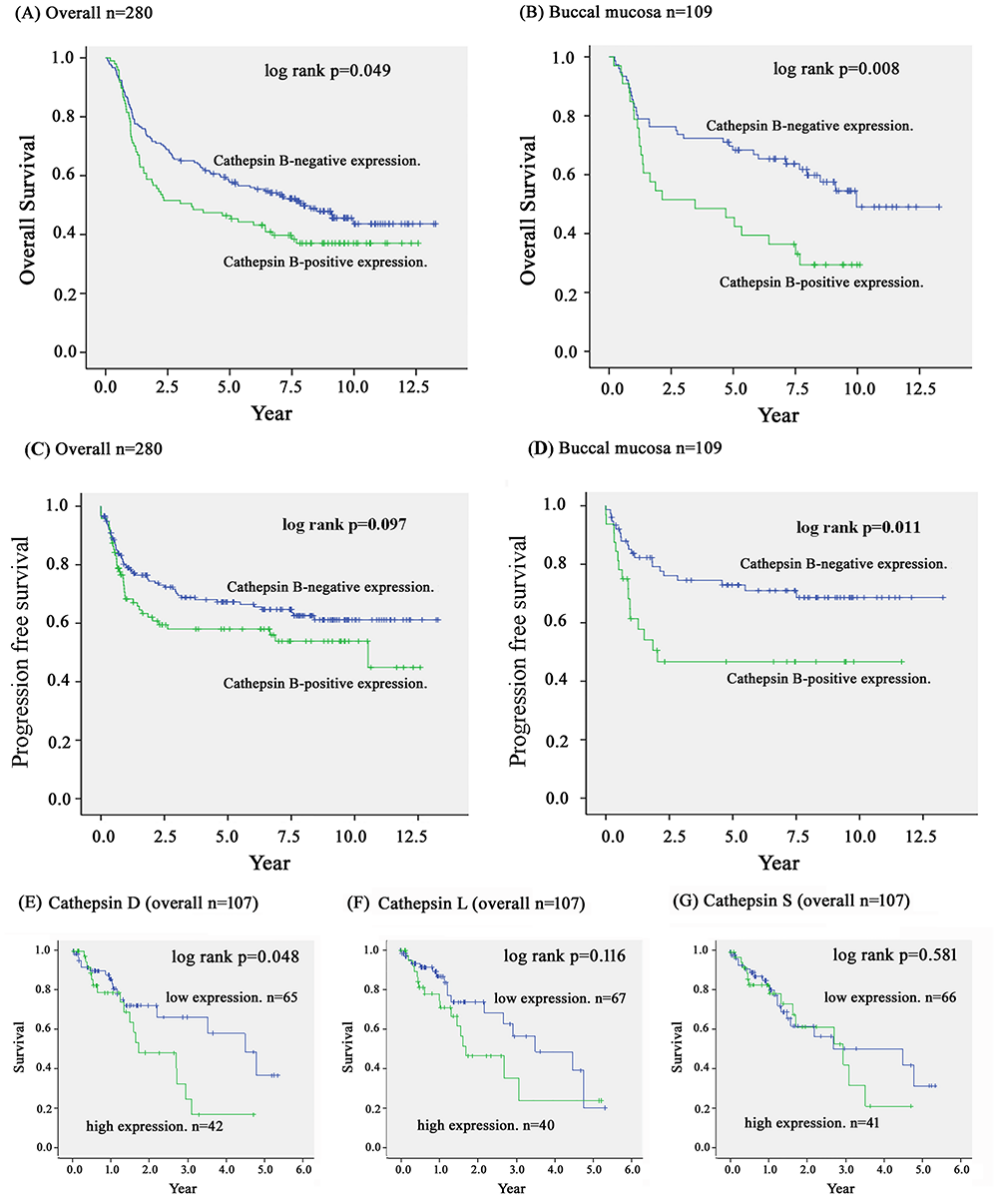
Kaplan-Meier survival curve showing the relation between cytoplasmic CTSB expression in primary tumors and survival in 280 oral squamous cell carcinoma (OSCC) patients (A) and 109 buccal mucosa squamous cell carcinoma patients (B). (C) The CTSB expression was not correlated with progression free survival in OSCC patients (p = 0.097). (D) The progression free survival of buccal mucosa squamous cell carcinoma patients with positive CTSB staining was significantly lower than that of patients with negative CTSB staining (p = 0.011). (E) From TCGA (The Cancer Genome Atlas) database (https://tcga-data.nci.nih.gov/tcga/), the Kaplan-Meier curve showed that higher expression of CTSD had a significantly poor survival in 107 oral cancer patients (p = 0.048). (F) The CTSL and (G) CTSS mRNA expression were not correlated with overall survival in oral cancer patients.

The Kaplan–Meier curve was used to evaluate the correlation of CTSB expression with overall survival and progression free survival. OSCC patients with CTSB expression had shorter overall survival than those without CTSB expression (Fig 2A). Buccal mucosa SCC patients with CTSB expression had significantly lower overall survival (p = 0.008) (Fig 2B). However, we did not find CTSB expression to be correlated with progression free survival in OSCC patients (p = 0.097) (Fig 2C) while CTSB expression had significantly lower progression free survival in the buccal mucosa SCC patients (p = 0.011) (Fig 2D).

Cathepsins family plays an important role in tumor progression [21]. Besides CTSB, other CTSs, such as cathepsin D (CTSD), cathepsin L (CTSL) and cathepsin S (CTSS), showed highly expression in invasive tumor and increase motility of cancer cells [22–24]. Therefore, the normalized RNA-Seq data of CTSD, CTSL and CTSS from 107 patients of three oral cancer anatomic subtypes (base of tongue, buccal mucosa and floor of mouth) from TCGA (The Cancer Genome Atlas) database (https://tcga-data.nci.nih.gov/tcga/) were selected for survival analyses. The Kaplan-Meier curve showed that oral cancer patients with higher expression of CTSD had a significantly poor survival in oral cancer patients (p = 0.048) (Fig 2E). However, expression of CTSL and CTSS was not associated with survival in oral cancer patients (Fig 2F and 2G).

**Fig 3:**
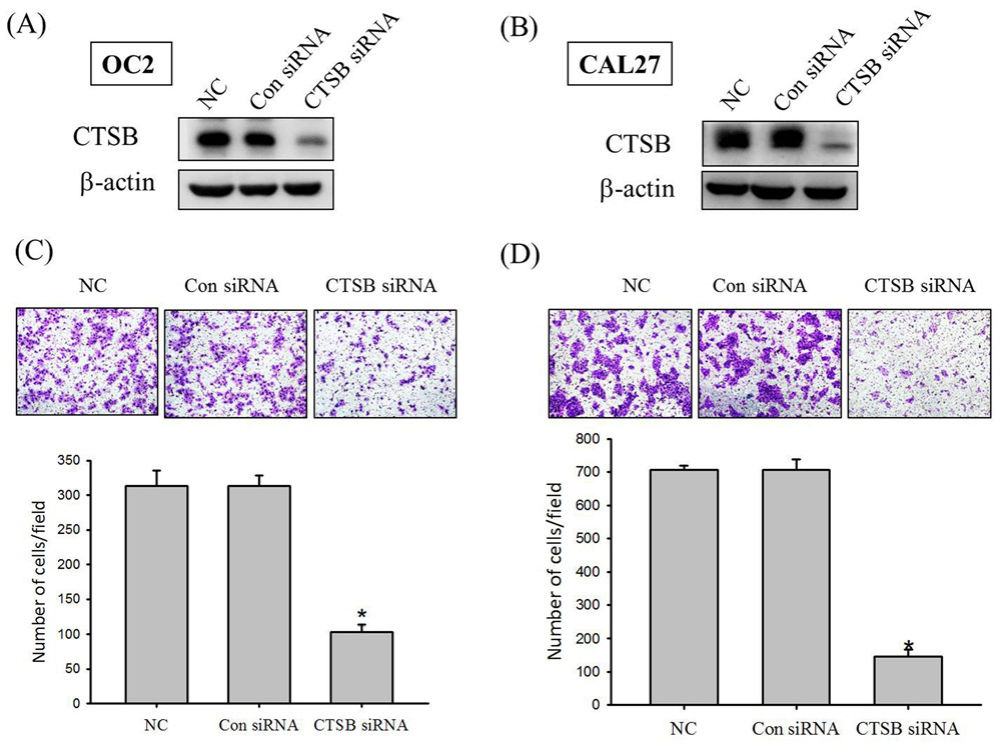
Cathepsin B knockdown in OC2 and CAL27 cells reduce cell migration. Western blot analysis showing the cathepsin B Knockdown efficiency in (A) OC2 cell and (B) CAL27 oral cancer cell lines. Detection of cell migration ability by transfected with control siRNA or CTSB siRNA in (C) OC2 cell and (D) CAL27 cell. Results shown that cathepsin B siRNA significantly reduced the migration of OC2 and CAL27 cells. *p<0.05.

Since we found that expression of CTSB was significantly correlated with the presence of lymph node metastasis, the effects of the CTSB knockdown on the oral cancer cell line were investigated by cell transwell migration assay. Compared with the control siRNA, results from Western blotting showed an approximately 45% reduction of CTSB expression after CTSB siRNA treatments in the OC2 and CAL27 cell lines (Fig 3A and 3B). Using the cell transwell migration assay, it was shown that CTSB siRNA significantly reduced the migration of OC2 and CAL27 cell lines (Fig 3C and 3D).

## Discussion

Oral cancer is still frequently diagnosed worldwide. In Taiwan and other neighboring countries, smoking and betel quid chewing are major risk factors and exert synergistic effects on oral cancer [2]. The prediction sites for intraoral carcinoma are the tongue and buccal mucosa. More than half of patients present with an advanced stage at their initial diagnosis, resulting in significantly high mortality. The tumor size, lymph node metastasis, and histological type are factors influencing the prognosis of oral cancer [25]. In this study, immunohistochemistry was used to evaluate CTSB expression in 280 OSCC patients. Higher CTSB expression was detected in patients with lymph node metastasis and poorly differentiated tumors. Moreover, CTSB expression in buccal mucosa carcinoma patients was significantly associated with a lower overall survival rate.

CTSB is one of the 12 human cysteine CTSs (B, C, F, H, L, K, O, S, V, W, X, and Z) and is expressed constitutively and associated with protein turnover in lysosomes [21]. It exhibits both endopeptidase and exopeptidase activities. The dual activity is attributed to structural changes in CTSB that are induced by the pH level of cells [26, 27]. Moreover, CTSB can regulate at different levels through posttranslational processing and trigger activation and inhibition. Various studies have demonstrated the causal role of CTSB in the multistep process of tumorigenesis. In CTSB-deficient mice, tumor cell proliferation was decreased, disrupting high-grade mammary carcinoma development [28]. Furthermore, CTSB can degrade the basement membrane and ECM for facilitating tumor progression [29]. CTSB knockdown in breast cancer cells may inhibit CTSB activity and attenuate ECM degradation through reduced type I collagen activity and bone metastasis *in vivo* [30]. Similarly, thyroid carcinomas with extracapsular invasion and metastasis that showed higher CTSB activity also exhibited higher type I and IV collagen degradation abilities [31]. In addition, lung cancer patients with upregulated CTSB tended to exhibit a higher rate of hematogenous and intrapulmonary metastasis [32]. CTSB expression in invasive tumors was positively correlated with lymphatic metastasis, suggesting that CTSB contributes to cervical cancer development [33]. Similar results were found in this study; cytoplasmic CTSB expression was correlated with positive lymph node metastasis and poorly differentiated tumors (Table 2).

Most studies have demonstrated that high CTSB expression is related to tumorigenesis in various human cancers. However, Guicciardi et al. indicated that CTSB may contribute to TNF-alpha-triggered apoptosis through the release of mitochondrial cytochrome c [34]. CTSB can reportedly cleave proapoptotic proteins, such as Bid, Bcl-2, and Bax [35]. Pratt et al. also demonstrated that CTSB is a positive regulator of the intrinsic apoptotic cascade. These findings imply that CTSB has two opposing effects on tumors: positive effects mediated by cleaving proapoptotic proteins and negative effects mediated by facilitating metastasis.

One limitation of the present study was the lacking of the information of smoking and betel quid used, which could provide additional support to our findings in this study. More detailed analysis based on amount and past history of betel nut and tobacco consumption may warrant further studies.

In this study, the role of CTSB expression in OSCC was clarified using immunohistochemical analysis of tissues from 280 OSCC patients. Our study demonstrated that CTSB expression was significantly associated with positive lymph node metastasis and higher tumor grade. The results also illustrated that OSCC patients with CTSB expression had lower overall survival and that CTSB can be used as a biomarker of overall survival in Taiwan.
